# Supplementary material for: Functional and Transcriptional Adaptations of Blood Monocytes Recruited to the Cystic Fibrosis Airway Microenvironment In Vitro
Source: Int J Mol Sci. 2021 Mar 3;22(5):2530. doi: 10.3390/ijms22052530 (PMC7959310; doi:10.3390/ijms22052530)
Supplement: Supplementary file 1 [file ijms-22-02530-s001.pdf]

FUNCTIONAL AND TRANSCRIPTIONAL ADAPTATIONS OF BLOOD MONOCYTES RECRUITED TO THE CYSTIC FIBROSIS AIRWAY MICROENVIRONMENT IN VITRO

Bijean D. Ford, Diego Moncada Giraldo, Camilla Margaroli, Vincent D. Giacalone,

Milton R. Brown, Limin Peng, Rabindra Tirouvanziam

SUPPLEMENTARY MATERIAL

Figures 6

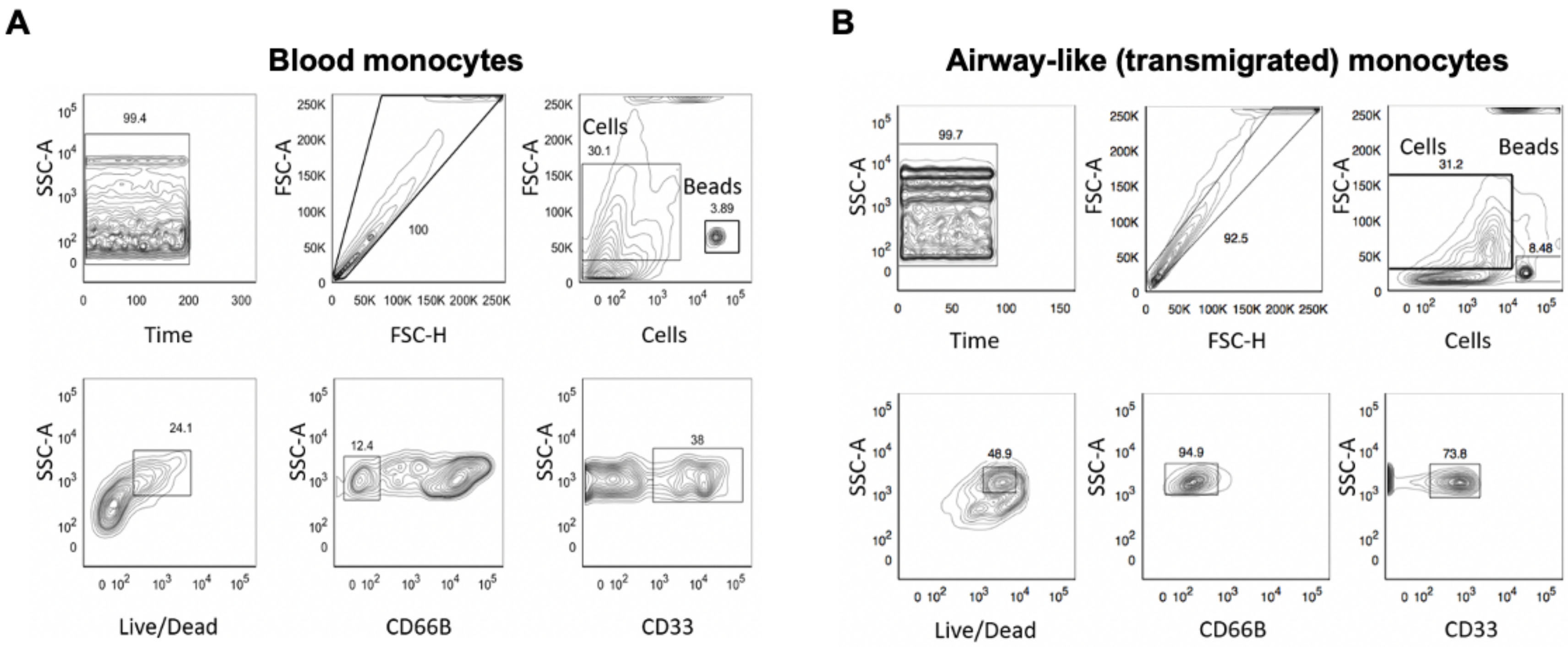

Figure S1. Flow cytometry gating. Shown are the sequential gates (top left to bottom right) used in our study to gate on **(A)** live blood monocytes pre-transmigration and **(B)** airway-like monocytes post-transmigration**.**

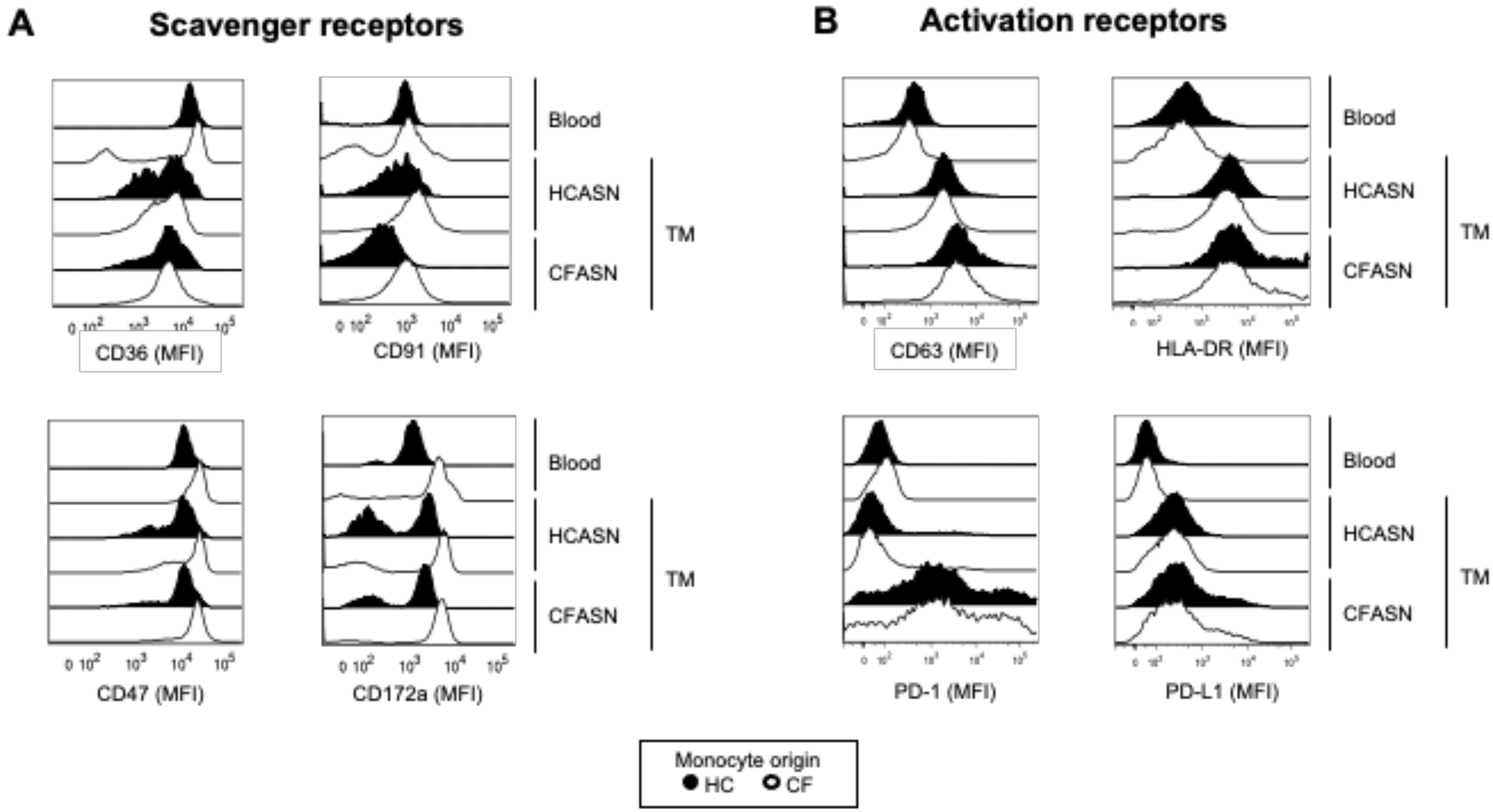

Figure S2. Flow cytometry histograms for chosen outcomes. Shown are representative histograms for gated monocytes from blood (HC, CF) and TM monocytes (HC, CF) recruited into HCASN or CFASN for **(A)** scavenger receptors and **(B)** activation receptors**.**

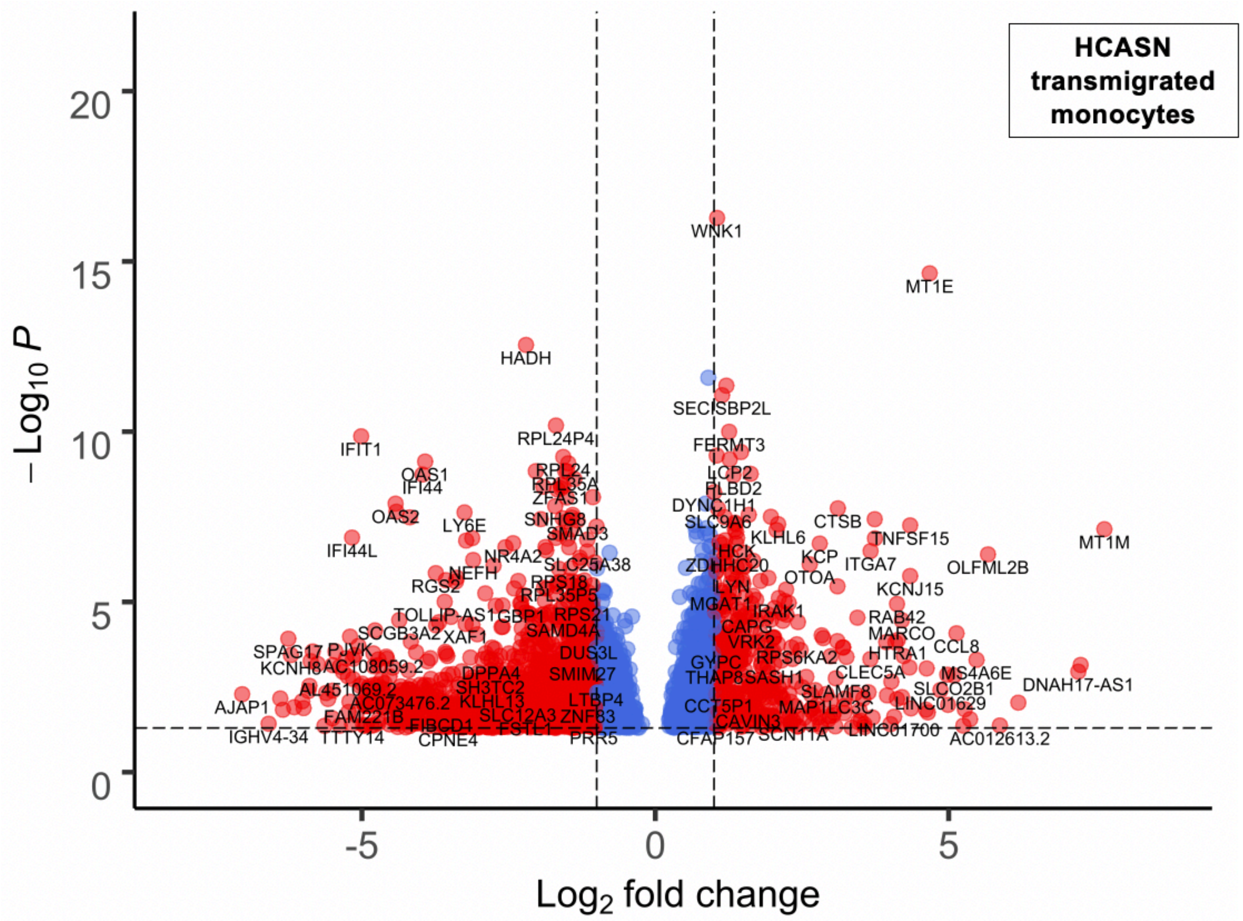

Figure S3. Transcriptomic analysis of HCASN-transmigrated monocytes. Volcano plot (log2 fold change greater or lower than 2 or -2, and a p-value less than 0.05; left) illustrating differentially expressed genes and pathways in monocytes transmigrated to HCASN compared to blood monocytes.

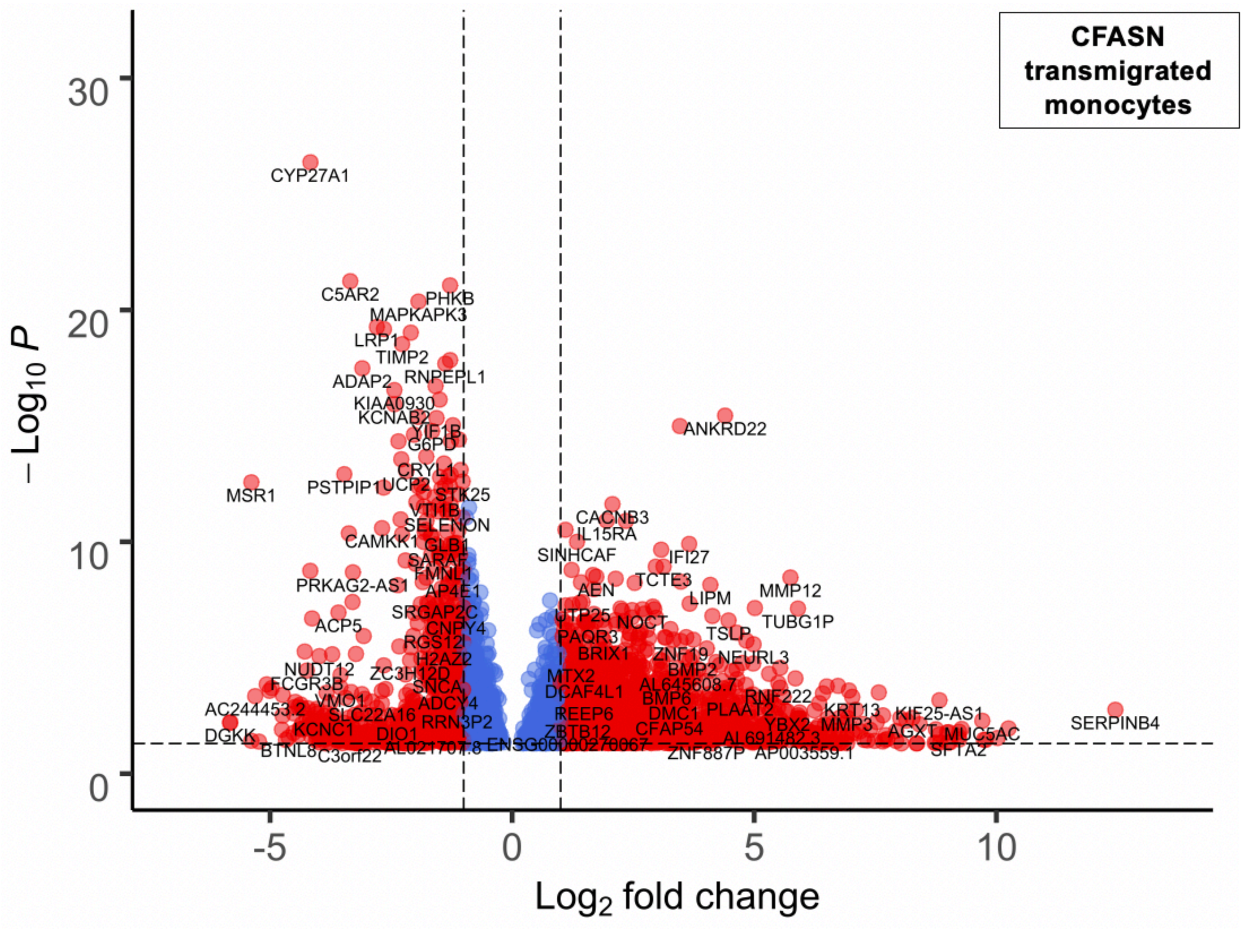

Figure S4. Transcriptomic analysis of CFASN-transmigrated monocytes. Volcano plot (log2 fold change greater or lower than 2 or -2, and a p-value less than 0.05; left) illustrating differentially expressed genes and pathways in monocytes transmigrated to CFASN compared to blood monocytes.

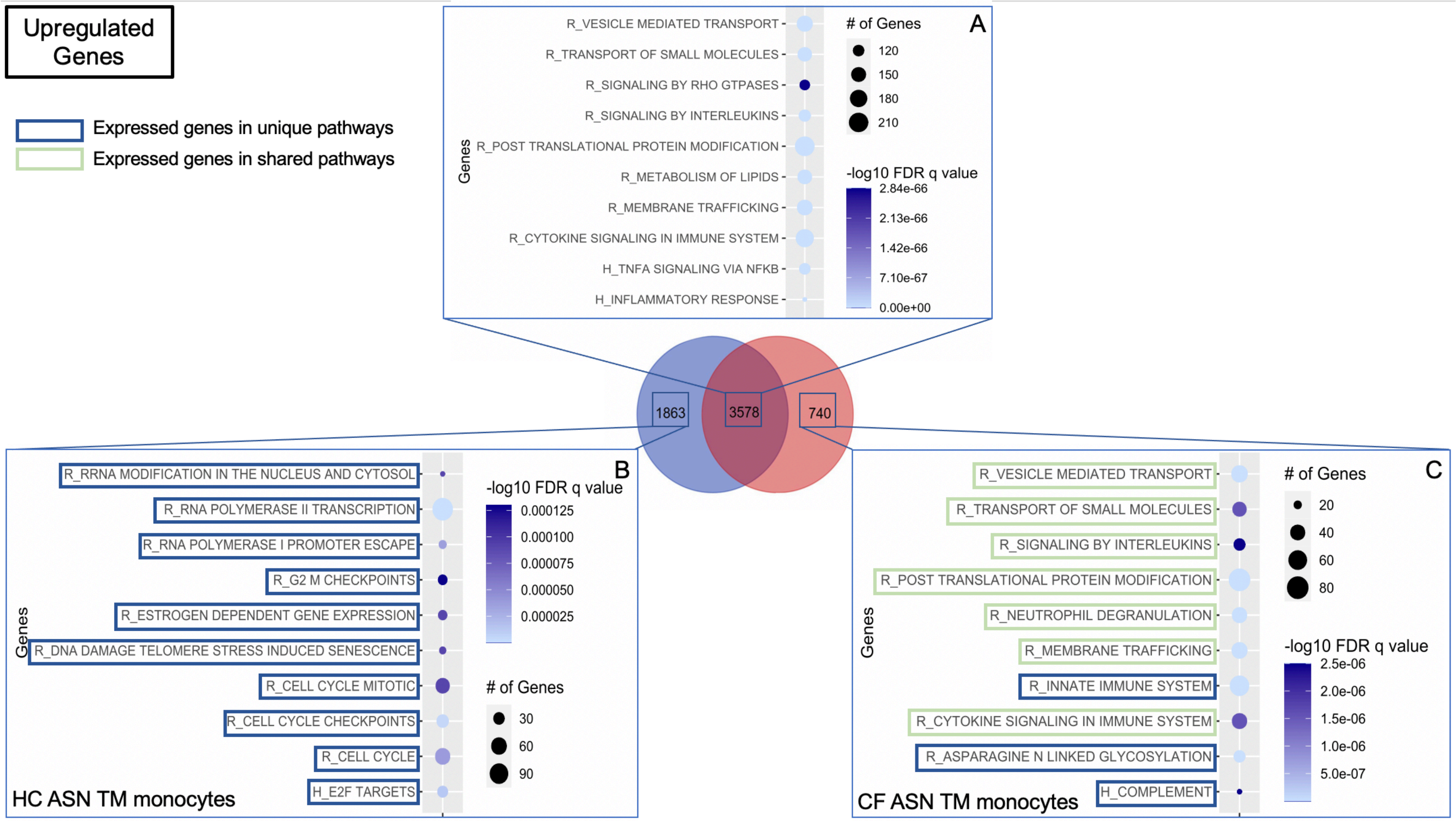

Figure S5. Pathway upregulation in HCASN-migrated and CFASN-migrated monocytes. The transcriptome of monocytes was analyzed at 10 hours post-transmigration towards HCASN or CFASN. Pathway enrichment analysis was conducted with MSigDB (Molecular Signatures Database v7. 2) using hallmarks, biocarta, reactome, and KEGG for the overlaps with our data sets, and FDR <0.05 on upregulated genes compared to blood monocytes. Shown are **(A)** shared upregulated pathways, and those upregulated more significantly in **(B)** HCASN-migrated monocytes, and **(C)** CFASN-migrated monocytes. Pathways are marked as H for hallmarks; K for Kegg; and R for reactome.

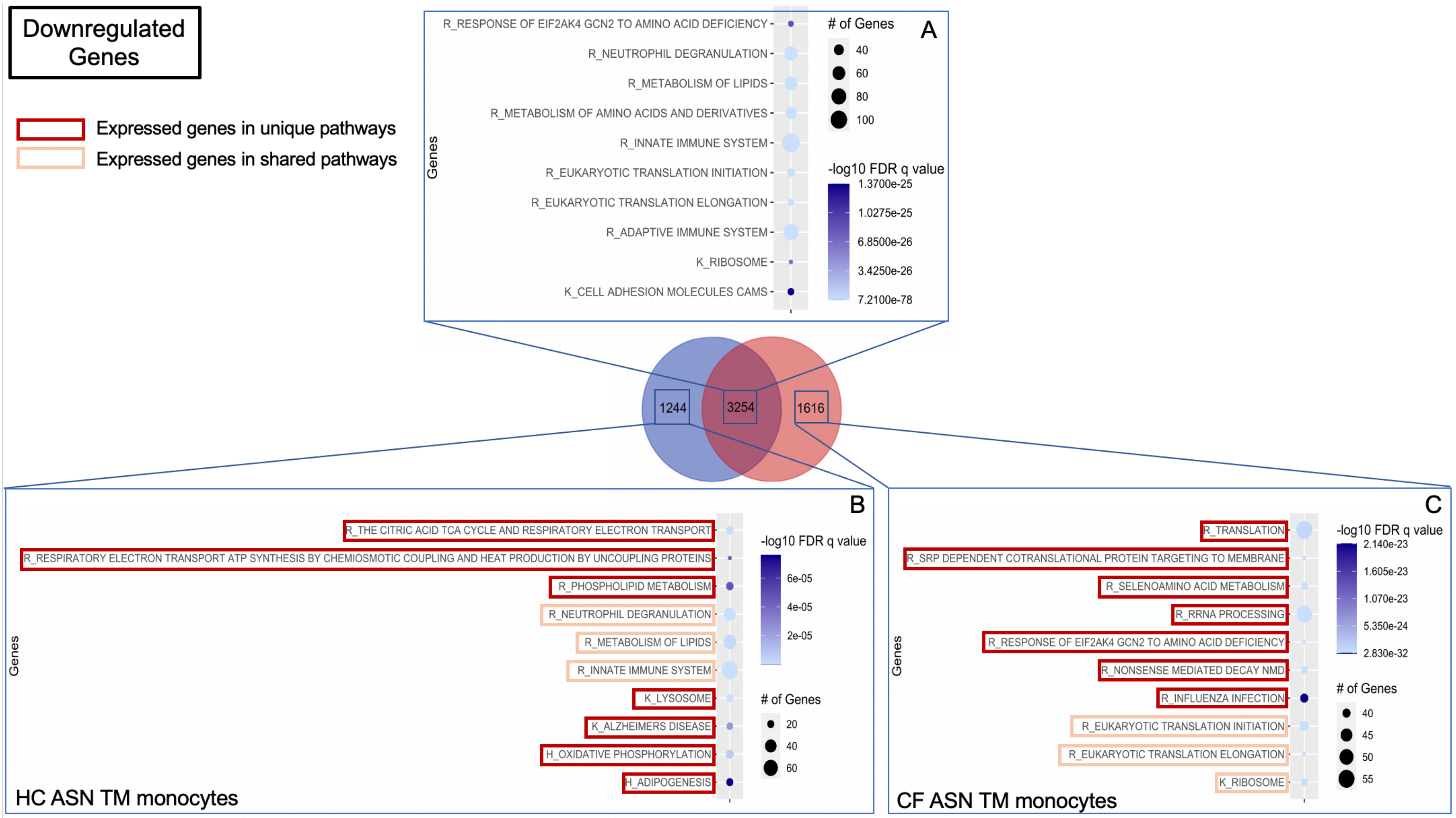

Figure S6. Pathway downregulation in HCASN-migrated and CFASN-migrated monocytes. The transcriptome of monocytes was analyzed at 10 hours post-transmigration towards HCASN or CFASN. Pathway enrichment analysis was conducted with MSigDB (Molecular Signatures Database v7. 2) using hallmarks, biocarta, reactome, and KEGG for the overlaps with our data sets, and FDR <0.05 on downregulated genes compared to blood monocytes. Shown are **(A)** shared downregulated pathways, and those downregulated more significantly in **(B)** HCASN-migrated monocytes, and **(C)** CFASN-migrated monocytes. Pathways are marked as H for hallmarks; K for Kegg; and R for reactome
